# Policy Implementation in the Digital Era: A Comprehensive Analysis of the Electronic-Based Government System (SPBE) in Indonesia

**DOI:** 10.12688/f1000research.175463.1

**Published:** 2026-04-15

**Authors:** Turino Yulianto, Choirul Saleh, Irwan Noor, Suryadi Suryadi

**Affiliations:** 1 Brawijaya University, Malang, East Java, Indonesia

**Keywords:** E-Government, Electronic-Based Government System, SPBE, Public Policy Implementation

## Abstract

**Purpose:**

The acceleration of digital transformation in the public sector has encouraged governments to adopt e-government systems to improve efficiency, transparency, and service quality. In Indonesia, this agenda is formalised through the Electronic-Based Government System (SPBE) under Presidential Regulation No. 95 of 2018. This study aims to analyse how SPBE is implemented in the Ministry of Manpower of the Republic of Indonesia as a central agency and in the Manpower Office of Banten Province as a regional agency, and to explain the dynamics and consequences of this implementation using public policy implementation theory.

**Methodology:**

A qualitative case study design is employed, combining in-depth interviews with policymakers, implementers, and users.

**Findings:**

The findings show that SPBE has driven the adoption of various digital applications and has begun to change work patterns from manual to digital in both central and regional offices. However, implementation is still characterised by fragmented systems, limited interoperability between central and local platforms, rigid digital procedures, uneven socialisation, and the persistence of manual routines. These patterns reveal gaps between formal policy standards and actual performance.

**Conclusion:**

The study implies that strengthening SPBE requires better integration between central and regional systems, design digital services that are simple and user-friendly, improve coordination and digital skills, and manage SPBE by involving many actors, not just through top-down instructions.

## 1. Introduction

In the digital era, governments across the world are increasingly expected to transform their bureaucratic structures and service delivery mechanisms through the use of information and communication technologies. ^1^ Digital government reforms are promoted as a means to improve efficiency, transparency, accountability, and citizen-centric public services. ^2^ Initiatives such as e-government, digital governance, and electronic-based public administration have thus become central pillars of administrative reform, particularly in developing countries. ^3^ However, implementing an digital policy agenda at various levels of government remains a complex and uncertain process. ^4^

In Indonesia, the Electronic-Based Government System ( *Sistem Pemerintahan Berbasis Elektronik*, SPBE) has been positioned as a key instrument to accelerate digital transformation in the public sector. ^5^ Presidential Regulation No. 95 of 2018 provides the normative framework for SPBE, outlining principles, standards, and governance arrangements intended to promote integrated, efficient, and interoperable digital systems across ministries, agencies, and subnational governments. The SPBE policy is expected to harmonise various legacy applications, reduce administrative burdens, and support better coordination in public service delivery. ^6^ Yet, the implementation of SPBE unfolds in a highly diverse administrative landscape, characterised by variations in institutional capacity, political commitment, infrastructure readiness, and human resources between central and local governments. ^7^ These conditions raise important questions about how the policy is actually implemented in practice and how it shapes the functioning of day-to-day public services.

Previous research on e-government and digital government in developing countries has generally focused on issues such as adoption factors, ^8, 9^ technological readiness, ^10, 11, 12^ user acceptance, ^13, 14^ and the impact of digitalisation on transparency and service quality. ^15, 16^ Many studies have examined single organisations or specific applications, often emphasising success stories or technical challenges. Fewer studies have offered a comprehensive, multi-level analysis of how a national digital government policy is interpreted and enacted across different tiers of government, and how policy implementation dynamics interact with existing institutional arrangements. In the Indonesian context, existing literature on SPBE tends to discuss regulatory frameworks, ^17^ technical architectures, ^18^ or sector-specific digital initiatives, ^19^ but systematic empirical analyses that connect national-level policy design, central–local relations, and implementation processes within concrete service domains remain limited.

Existing studies on SPBE implementation in Indonesia largely rely on classical, linear models of policy implementation, typically framed in top-down or bottom-up terms and focused on actor compliance with regulations. ^20, 21^ Empirical analyses are frequently confined either to the national level or to single local case studies, with limited comparative evidence on how implementation unfolds across central and subnational governments. Moreover, relatively few studies explicitly examine public service inefficiency as a key outcome of SPBE implementation, or capture the perspectives of multiple actor groups, including policy-makers, implementers, and users. As a result, there remains a significant gap in the literature regarding multi-level, multi-actor analyses of SPBE that integrate insights from classical policy implementation theory to explain how fragmented bureaucratic structures, limited coordination, and centre–province asymmetries shape implementation outcomes, including unintended inefficiencies in public service delivery. ^22^

This study aims to provide a comprehensive analysis of SPBE policy implementation in Indonesia by using the labour sector as a representative case. It examines how SPBE, as mandated by Presidential Regulation No. 95/2018, is interpreted, implemented, and experienced at the Ministry of Manpower at the national level and the Provincial Manpower Office of Banten at the subnational level. Specifically, the article seeks to: (1) map how the national regulatory and institutional framework of SPBE is translated into organisational arrangements, digital architectures, and core applications in the labour sector; (2) analyse how actors at the central and provincial levels interpret and enact SPBE in practice, including the role of organisational capacity, technological and human resource readiness, and inter-organisational coordination in shaping implementation; and (3) explain the emerging patterns of integration, fragmentation, and inefficiency in SPBE implementation, using public policy implementation theory to assess their implications for public service delivery in the labour sector. By using this theoretical perspectives with a multi-level empirical analysis, the article contributes to the growing literature on digital government in developing countries and offers practical insights for strengthening the design, coordination, and governance of SPBE in Indonesia.

## 2. Literature review

### 2.1 Theoretical background: Public policy implementation

Public policy implementation theory seeks to explain how formal policy decisions are translated into actions and outcomes within administrative systems. ^23, 24^ Early work in this field distinguishes between top down approaches, which emphasise the authority of central decision makers and the design of control mechanisms, and bottom up approaches, which highlight the discretion, strategies, and coping behaviours of local implementers and street level bureaucrats. ^20, 21^ Classical implementation frameworks generally identify a set of intervening variables that connect policy goals to outcomes, such as the clarity and consistency of objectives, the availability of resources, the characteristics of implementing organisations, interorganisational communication and enforcement, and the socio political context. ^20^ These perspectives have been widely used to analyse public sector reforms in developing countries and provide a structured vocabulary for examining why similar policies may produce different results across settings.

In this study, public policy implementation theory used to analyse the implementation of the Electronic Based Government System (SPBE) in Indonesia. Implementation theory provides the core categories for examining policy content, institutional arrangements, resources, organisational characteristics, and actor dispositions that shape how SPBE is formally designed and operationalised across government levels. This theoretical background informs the analysis of how SPBE is implemented and how these implementation dynamics influence public service delivery outcomes.

### 2.2 E-Government

E-government is commonly understood as the application of information and communication technologies (ICTs) to improve the accountability, efficiency, and effectiveness of public organisations in delivering public services. ^25^ E-government can be defined as the use of information technologies by government agencies to transform their relations with citizens, businesses, and other arms of government. ^26^ It seeks to achieve better service delivery and improved interactions with business and industry. E-government also aims to promote citizen empowerment through access to information and to support more efficient government management. ^16^ These efforts are expected to yield benefits such as reduced corruption, increased transparency, greater convenience, revenue growth, and cost reductions. E-government can also be seen as the effective and efficient use of ICT to provide government services to citizens and businesses, enhance stakeholder satisfaction, and promote participation by giving citizens easy online access to information and services. ^2^

At the global level, the development of e-government is often analysed through maturity models and composite indicators. ^27^ Zhang and Kimathi ^28^ has identified a series of stages of e-government development, ranging from the provision of basic information, to enhanced interaction, transactional services, and fully integrated and connected services that support participation in decision-making. Many developing countries still struggle to reach the transactional and fully integrated stages because of constraints related to infrastructure, human resources, and institutional capacity. ^29, 30^ The UN E-Government Development Index (EGDI) shows gradual global improvement, with an increasing number of countries in the high and very high EGDI categories and positive correlations between EGDI scores and outcomes such as better SDG performance, lower corruption perception, and higher foreign direct investment.

In Indonesia, e-government has been promoted through the e-Indonesia initiative, which aims to improve public services, close the digital divide, reduce corruption through transparency, enhance education quality, support economic growth, and improve citizens’ quality of life. ^22^ To realise these goals, the government has adopted programmes on open government, human resource development, ICT infrastructure investment, citizen participation, and institutional and policy development. ^31, 32^ However, empirical assessments indicate that Indonesia, like many other developing countries, still faces challenges in fully developing e-government; for example, only a relatively small proportion of e-government services have been found to be fully accessible and functional, and implementation gaps remain between policy design and practice. ^22^

### 2.3 Electronic-based government system (SPBE)

SPBE is Indonesia’s national framework for digital government reform. ^33^ Presidential Regulation No. 95 of 2018 defines SPBE as an instrument to realise clean, effective, transparent, and accountable governance, as well as high-quality and trustworthy public services through the integrated use of information and communication technologies. ^34^ SPBE consolidates earlier e-government initiatives that were regulated through a series of laws and presidential decrees on electronic information, public information disclosure, electronic transactions, public services, and bureaucratic reform. Before the issuance of Presidential Regulation No. 95 of 2018, e-government policies tended to be scattered and sectoral, focusing on technical or legal aspects rather than offering an integrated national framework for cross-government interoperability. ^35^

Presidential Regulation No. 95 of 2018 is therefore widely regarded as a major milestone in Indonesia’s digital bureaucracy agenda. The regulation sets out SPBE’s vision, mission, objectives, targets, governance, architecture, interoperability, and national evaluation mechanisms, while assigning clear roles and responsibilities to ministries, central agencies, and subnational governments. It is closely linked to the broader e-Indonesia roadmap, which emphasises improving public services, closing the digital divide, reducing corruption through transparency, enhancing education quality, fostering economic growth, and improving citizens’ quality of life. ^22^

At the same time, the literature on SPBE and e-government in Indonesia highlights persistent implementation challenges. Studies note the proliferation of fragmented applications across institutions, limited interoperability and data integration, uneven ICT infrastructure, and disparities in digital capability among civil servants and regions. ^36^ Evaluations also suggest that SPBE monitoring is often compliance-oriented and document-based rather than focused on real-time performance and adaptive learning. These persistent challenges indicate a need for more in-depth, multi-level analyses of SPBE implementation that move beyond normative and technical assessments to examine how it operates in concrete administrative and service delivery contexts.

## 3. Methods

### 3.1 Research design

This study employs a qualitative approach with a case study design. A case study is understood as an in-depth empirical inquiry that examines a contemporary phenomenon within its real-life context, particularly when the boundaries between the phenomenon and its context are not clearly evident. ^37^ This design is appropriate for gaining a deep understanding of how the SPBE policy is implemented within the Indonesian bureaucracy by examining implementation dynamics, coordination among actors, and implications for public service governance. The case study approach allows the use of multiple data sources, including interviews, observations, and documents, so that implementation processes can be examined in their administrative and institutional context.

### 3.2 Study sites and focus

The empirical focus is the implementation of the Electronic-Based Government System (SPBE) as mandated by Presidential Regulation No. 95 of 2018 in the labour sector. Two sites were selected to capture both central and provincial perspectives:

1) The Ministry of Manpower of the Republic of Indonesia (Kemnaker RI) in Jakarta, particularly units related to SPBE such as the Data and Information Technology Centre, the Organisation Bureau, and directorates responsible for labour-related services.
2) The Provincial Manpower Office of Banten, especially units responsible for labour market information and digital service delivery.

The study concentrates on three main aspects: (1) the implementation of key SPBE-related applications in the labour sector, including SISNAKER, WLKP Online, SIPK, and online training and certification services; (2) the extent to which implementation is aligned with the mandates of Presidential Regulation No. 95 of 2018; and (3) factors that contribute to fragmentation, overlapping applications, bureaucratic resistance, and inefficiencies in public service delivery.

### 3.3 Participants and data collection

Participants were selected using purposive sampling combined with criterion-based selection, targeting individuals who possess relevant knowledge and direct involvement in SPBE implementation in the labour sector. The study involves three categories of actors at each level of government: policy-makers, implementers, and users of SPBE-related systems. At the central level, participants include senior officials in charge of data and information management, ICT staff, and functional officials who use SPBE applications in their daily work. At the provincial level, participants include the head of the provincial manpower office, mid-level managers responsible for planning and placement of labour, and staff who operate SPBE-based services. Additional informants were identified through snowball sampling until data saturation was reached, indicated by the repetition of information and the absence of new themes.

Data were collected through several complementary techniques. First, semi-structured in-depth interviews were conducted with central and provincial officials, technical staff, and functional officers to explore their experiences, perceptions, and assessments of SPBE implementation. Second, non-participant observation was carried out to examine how SPBE applications are accessed and used in practice, including user interaction with systems and the handling of technical and procedural constraints. Third, document analysis was undertaken on key regulatory documents, SPBE guidelines, evaluation reports, and internal data from Kemnaker RI and the Banten Provincial Manpower Office. In some instances, focused group discussions with representatives of employers and workers were used to gather user perspectives on SPBE-based services.

**Informed consent and confidentiality.** Prior to each interview and discussion, participants were provided with an information sheet outlining the study objectives, procedures, expected duration, potential risks and benefits, confidentiality safeguards, and their right to decline or withdraw at any time without consequences. Verbal informed consent was obtained from all participants before data collection commenced. Verbal consent was selected instead of written consent to minimise administrative burden and reduce potential discomfort or perceived institutional risk among participants, particularly policymakers and public officials involved in governance discussions. The use of verbal informed consent was reviewed and approved by the Ethics Committee of the Faculty of Administrative Sciences, Universitas Brawijaya (Approval No. 10406/UN10.F0301/B/PP/2025). To protect privacy, no personally identifying information was recorded in the research database; transcripts and field notes were anonymized, and all files were stored securely with restricted access.

### 3.4 Data analysis

Data analysis followed the interactive model proposed by Miles et al., ^38^ which involves three interrelated processes: data reduction, data display, and conclusion drawing and verification. Data reduction was carried out by organising interview transcripts, observation notes, and documents into thematic categories related to policy design, organisational and inter-organisational coordination, technological and human resource capacity, fragmentation of systems, and service delivery outcomes. Data display took the form of matrices, comparative tables between central and provincial levels, and thematic narratives that facilitated the identification of patterns and relationships across cases. Conclusions were developed iteratively and continuously checked against the data as new information was incorporated.

The validity of the findings was enhanced through method and source triangulation. Method triangulation was achieved by comparing information from interviews, observations, documents, and group discussions. Source triangulation involved cross-checking information obtained from different categories of actors at both central and provincial levels, as well as comparing these accounts with official records and archival materials related to SPBE implementation. Where necessary, follow-up communication with key participants was undertaken to clarify interpretations and reduce the risk of misrepresentation.

## 4. Results

### 4.1 Regulatory framework of SPBE

The Electronic-Based Government System ( *Sistem Pemerintahan Berbasis Elektronik*, SPBE) is Indonesia’s national framework for organising government administration through the use of information and communication technologies to deliver public services. ^33^ Presidential Regulation No. 95 of 2018 on SPBE defines it as an instrument to realise clean, effective, transparent, and accountable governance, as well as high-quality and trustworthy public services through integrated ICT. ^34^ The regulation applies to ministries and central agencies, as well as provincial and district or municipal governments, and introduces national arrangements for SPBE architecture, governance, and evaluation.

Presidential Regulation No. 95 of 2018 consolidates a series of earlier, more fragmented e-government regulations. Before 2018, policies on electronic information, electronic transactions, public information disclosure, public services, financial information systems, and bureaucratic reform were largely sectoral and technical, without a single overarching framework for interoperability and coordinated digital governance. SPBE brings these strands together in a comprehensive and cross-sectoral regulation that defines a national architecture, clarifies roles and responsibilities, and establishes an annual SPBE evaluation system. Given that SPBE consolidates previously fragmented e-government mandates into a single national framework, Table 1 presents the main regulations that provide the policy and operational foundation for SPBE.

**Table 1.** summarises selected key regulations for SPBE.

No	Regulation	Main content/relevance
1	Presidential Instruction No. 3 of 2003 on National Policy and Strategy for E-Government Development	First formal regulation on e-government; required each institution to prepare its own e-government plan, but did not regulate cross-institution integration.
2	Law No. 11 of 2008 on Electronic Information and Transactions	Provided the legal basis for electronic services and transactions, including in government; focused on the legality of electronic documents and digital transactions.
3	Government Regulation No. 82 of 2012 on the Implementation of Electronic Systems and Transactions	Regulated the security and management of electronic systems in public and private sectors; relevant for data centres, information security, and system audits.
4	Presidential Regulation No. 81 of 2010 on the Grand Design of Bureaucratic Reform 2010–2025	Linked the use of ICT to bureaucratic reform, efficiency, and accountability, but without detailed technical arrangements for SPBE.
5	Regulation of the Minister of Administrative and Bureaucratic Reform No. 41 of 2010 on Guidelines for E-Government Development	Provided guidelines for the development of ICT-based government services, but remained sectoral and not bound by a national architecture.
6	Presidential Regulation No. 95 of 2018 on the Electronic-Based Government System (SPBE)	First comprehensive and cross-sectoral regulation on SPBE; establishes national architecture, governance, interoperability, and a national SPBE evaluation system.

Compared with previous instruments, Presidential Regulation No. 95 of 2018 represents a qualitative shift in how digital bureaucracy is regulated. It moves from fragmented, project-based e-government initiatives towards an integrated, system-wide approach to digital governance. Table 2 summarises the main advantages of Presidential Regulation No. 95 of 2018 compared to the pre-SPBE regulatory landscape.

**Table 2.** Key Advantages of presidential regulation no. 95 of 2018.

No	Aspect	Before rresidential regulation 95/2018	After presidential regulation 95/2018
1	Policy approach	Fragmented, project-based, and sector driven	Integrated and systemic, based on a national SPBE architecture
2	Type of regulation	Circular letters, presidential instructions, and sectoral policies	Presidential Regulation with higher legal status and cross-sector coverage
3	Vision and objectives	Focus on specific e-government applications or online services	Building an integrated, efficient, and accountable digital government
4	Regulatory scope	Limited to ICT or data within individual sectors	Covers services, governance, internal policies, human resources, budgeting, and risk management
5	Architecture and technical standards	No binding national architecture for systems and data	National SPBE Architecture established to ensure interoperability and efficient system development
6	Inter-institutional coordination	Limited coordination and weak synergy among institutions	National SPBE Coordination Team and SPBE management units at central and local levels
7	Monitoring and evaluation	No unified national e-government evaluation system	National SPBE evaluation system linked to the SPBE index, conducted regularly
8	Focus of digital transformation	E-government as an administrative support tool	SPBE as a driver of governance transformation towards an adaptive digital bureaucracy

Since 2018, the implementation of Presidential Regulation No. 95 of 2018 has been monitored annually through the national SPBE index. The index assesses the maturity of SPBE implementation across ministries, central agencies, and local governments based on a set of standardised indicators. ^39^ These annual evaluations provide a key reference point for understanding how the regulatory framework is translated into practice and form an important empirical basis for the analysis in the following sections.

### 4.2 Implementation of SPBE at the central level

The Ministry of Manpower of the Republic of Indonesia is a key ministry that manages labour affairs for millions of workers, both domestic and migrant. ^40^ In this context, the ministry is expected to become one of the leading institutions in implementing SPBE in its daily operations. Institutionally, SPBE implementation is coordinated by the Data and Information Centre (Pusdatin) under the Secretariat General, whose mandate has been strengthened through internal ministerial regulations on data governance, applications, and SPBE architecture. ^41^ In the national SPBE index issued annually by the Ministry of Administrative and Bureaucratic Reform for 2020–2023, the Ministry of Manpower has consistently achieved scores in the “good” category (approximately 2.6–3.0), but has not yet reached the “very good” category.

Interview data at the central level show that different actor groups hold different understandings of SPBE, as summarised in Table 3. Policy-makers tend to view SPBE as an organisational architecture and management tool, with a focus on regulations and integration across units. Implementers emphasise the practical challenges of building architecture, integrating data and services, and coordinating across ministries and agencies. Users, by contrast, mainly experience SPBE through specific applications such as digital attendance systems, the *Srikandi* document system, and budgeting applications that have become embedded in daily work routines. Across these perspectives, several common obstacles emerge, including internal fragmentation between units, sectoral ego, limited inter-operability with other institutions, and an implementation orientation that often prioritises compliance with index requirements and documentation rather than substantive digital transformation.

**Table 3.** Summary of central-level actor perspectives on SPBE.

Aspect	Policy-makers	Implementers	Users
Understanding of SPBE	SPBE as organisational architecture and management tool	Initially seen as an IT issue, then as cross-unit business process and data architecture	SPBE understood mainly as specific applications used in daily work
Main implementation focus	Meeting policy and index requirements, building internal regulations and integration	Strengthening architecture, data integration, and internal rules on data protection and systems	Using digital services such as attendance, document management, and budgeting in routine work
Key obstacles	Fragmentation between units, sectoral ego, different priorities, index-oriented implementation	Sectoral ego, difficult inter-agency coordination, limited inter-operability and resources	Rigid systems, uneven communication, resistance among less tech-literate staff
Perceived change	Limited transformation due to administrative focus	Some progress in integration, but fragmentation remains strong	Clear shift from manual to digital work, but with adaptation challenges
Suggestions	SPBE should become an organisational agenda, not only a technical project	Need a strong leading sector and simplified coordination mechanisms	Systems should be more user-friendly, flexible, and equipped with fair correction mechanisms

At the same time, central-level actors report significant changes in work practices, particularly the shift from manual to digital processes, even though some staff remain resistant and systems are sometimes perceived as rigid or not user-friendly. Technical constraints such as server capacity and the absence of flexible correction mechanisms, for example in attendance recording, also affect user acceptance. The Covid-19 pandemic increased pressure for digitalisation and intensified the use of SPBE-related systems, ^42^ but did not automatically translate into a qualitative leap in SPBE implementation. In summary, SPBE implementation at the Ministry of Manpower shows clear progress in digitalising core processes, yet remains constrained by institutional fragmentation, uneven capacities, and a strong administrative and index-oriented approach to implementation.

### 4.3 Implementation of SPBE at the provincial level

At the provincial level, this study focuses on the implementation of SPBE in the Banten Provincial Manpower Office. The findings indicate a significant gap between the national SPBE policy framework and local-level understanding and practice. Although Presidential Regulation No. 95 of 2018 has been in force for more than five years, senior provincial officials are generally familiar with SPBE only in broad terms, and often perceive it as an administrative instruction rather than a comprehensive digital transformation agenda. In practice, SPBE implementation in Banten has been driven by local initiatives, most notably the development of the *SiLoker* application, an Android-based platform designed to connect job seekers and companies free of charge.

Table 4 summarises provincial-level actor perspectives on SPBE at the Banten Provincial Manpower Office. From the perspective of policy-makers and implementers in the provincial office, *SiLoker* represents a concrete local innovation that responds to regional needs and is based on relevant ministerial regulations. However, the development and utilisation of SiLoker are constrained by limited budgets, uneven ICT capacity, ageing infrastructure, and strong sectoral ego across local government agencies. The application is not yet fully integrated with national systems such as *SiapKerja* or with systems in districts and municipalities. Coordination with other local agencies, such as the regional planning and communication offices, is carried out but often hindered by differing priorities, institutional silos, and changing leadership.

**Table 4.** Summary of provincial-level actor perspectives on SPBE.

Aspect	Policy-makers	Implementers	Users
Understanding of SPBE	Aware of SPBE and Presidential Regulation 95/2018 in general, but limited substantive understanding	Understand SPBE mainly through ministerial regulations related to labour services	Know SPBE through daily use of the SiLoker application
Main implementation focus	Local digital initiatives such as SiLoker, constrained by budgets and HR	Development and operation of SiLoker to match job seekers and companies	Use of SiLoker to disseminate vacancies and support recruitment
Key obstacles	Limited budget and ICT staff, sectoral ego, dependence on leadership	Limited socialisation, low familiarity among many companies, lack of full integration with national and district systems	Incomplete socialisation, companies not fully committed, limited political support
Perceived change	Digital initiatives exist but are vulnerable to leadership changes and lack sustainability	Digital services make labour intermediation more transparent, but remain partial and fragmented	Clear benefits in access to information and efficiency for job seekers and companies
Suggestions	Clear national architecture with room for local adaptation and stronger orchestration	Stronger socialisation, budget support, and technical integration with national systems	Stronger political and regulatory support from regional leaders to encourage wider adoption

Users at the provincial level report that SiLoker has brought tangible benefits for job seekers and companies by widening the reach of vacancy information from limited local channels to the entire province and by reducing opportunities for intermediaries and informal fees. The application is used frequently by job seekers, and companies appreciate that it is free and transparent. Nevertheless, adoption remains uneven because not all companies are aware of or confident in the system, and ongoing socialisation efforts are constrained by limited resources. Users also emphasise the importance of stronger political backing from regional leaders, particularly the governor, to encourage companies to use SPBE-based services more systematically. Overall, SPBE implementation in Banten Province is characterised by partial digital innovation in labour services, persistent fragmentation, dependence on leadership and local initiatives, and significant gaps between normative policy, institutional capacity, and everyday practice.

### 4.4 Comparative analysis of SPBE implementation at the central and provincial levels

At the central level, SPBE implementation is supported by a relatively strong regulatory basis and a wide range of internal applications, such as digital attendance systems, the *Srikandi* document platform, and budgeting systems. Policy-makers tend to interpret SPBE as an organisational architecture and management tool that should integrate processes, data, and services across units. However, implementers and users report internal fragmentation, overlapping systems, limited inter-operability, and an implementation approach that is often oriented towards meeting SPBE index requirements and documentation. Users frequently experience confusion due to the number of applications, uneven socialisation, and rigid procedures, for example the absence of correction mechanisms in digital attendance systems. Table 5 presents a comparative summary of SPBE implementation perspectives at the central and provincial levels.

**Table 5.** Comparative perspectives on SPBE implementation at central and provincial levels.

Actor	Central level	Provincial level
Policy-makers	View SPBE as organisational architecture and management tool, supported by a strong national regulatory framework but complicated derivative regulations and overlapping internal rules	Aware of SPBE regulations in general, but understanding is partial. Local applications are developed mainly to respond to national requirements and local needs
Implementers	Focus on building architecture, integrating data and services, and coordinating across units. Face internal fragmentation, sectoral ego, limited infrastructure, and challenging inter-agency coordination	Develop and operate SiLoker as a concrete SPBE initiative. Constrained by limited socialisation, budgets, ICT capacity, and incomplete integration with national and district systems
Users	Use multiple applications in daily work (attendance, documents, budgeting) but often feel overloaded and confused due to many systems, limited socialisation, and rigid procedures	Use SiLoker frequently and perceive clear benefits in access and efficiency, but adoption remains uneven and depends on political and organisational support

At the provincial level, SPBE implementation appears more directly connected to immediate service needs through local innovations such as the SiLoker application, which links job seekers and companies free of charge. Policy-makers and implementers in Banten emphasise the practical benefits of SiLoker for labour intermediation, and users report clear improvements in access to vacancy information and efficiency in recruitment processes. Nonetheless, implementation is constrained by limited budgets, uneven ICT capacity, weak integration with national and district systems, and dependence on political and organisational support. Socialisation is incomplete, many companies are still unfamiliar or hesitant to use the system, and changes in leadership can threaten the continuity of digital initiatives.

Overall, the comparison shows that central-level implementation is strong in regulatory authority but faces challenges in internal coordination and user adaptation, while provincial implementation is stronger in local innovation and perceived service benefits but weaker in political support, resources, and system integration. These differences point to multi-level fragmentation in SPBE implementation, encompassing normative, structural, and communicative dimensions. Such fragmentation contributes to inefficiencies, for example central staff struggling to navigate multiple applications and provincial companies underutilising government digital platforms.

## 5. Discussion

### 5.1 SPBE Implementation from the perspective of public policy implementation theory

Public policy implementation theory provides a useful framework for understanding why the SPBE policy, although normatively clear, still produces various forms of inefficiency in practice. ^20, 21^ The SPBE framework as mandated by Presidential Regulation No. 95 of 2018 explicitly aims to improve the efficiency, transparency, and accountability of public services. However, the empirical findings from the Ministry of Manpower and the Banten Provincial Manpower Office indicate that these objectives are only partially understood and internalised by implementers at different levels. By drawing on the public policy implementation theory, the analysis reveals that gaps in goal clarity, implementation structure, resources, communication, and contextual alignment all contribute to the persistence of inefficiencies in SPBE implementation.

From a top down perspective, Sabatier and Mazmanian ^20^ emphasise the importance of clear policy objectives, a coherent implementation structure, and supportive socio political conditions. In the case of SPBE, the formal goals are clearly articulated in the presidential regulation, yet field interviews show that these goals are not fully understood by local actors. A key official in the Banten Manpower Office, for example, reported having heard of the regulation but not having examined its substance in detail, which suggests that the intended objectives of SPBE have not been thoroughly internalised at the provincial level. Structurally, implementation relies on a combination of central applications, such as SiapKerja, and local systems, such as SiLoker, but technical and institutional integration between these platforms remains weak. At the same time, resistance from staff at the centre and limited socialisation in the regions indicate that the political administrative environment is not fully conducive to consistent top down steering. In this view, SPBE related inefficiencies are a consequence of incomplete transmission of objectives, fragmented implementation arrangements, and less than favourable contextual conditions.

Van Meter and Van Horn ^21^ process model further clarifies how standards, resources, interorganisational communication, and implementer dispositions shape SPBE outcomes. Standard operating procedures for SPBE, such as the mandatory use of *Srikandi* for correspondence and geo tagged e-attendance systems, are formally defined. However, these standards can be overly rigid and generate inefficiencies when they do not provide mechanisms to accommodate reasonable corrections, for example when staff forget to clock in despite actually being present at work. Resource constraints are particularly visible at the provincial level, where limited budgets restrict socialisation activities and prevent wider dissemination of SiLoker to the large number of firms operating in Banten. Communication and coordination between central and local agencies are also uneven, which contributes to overlapping initiatives and uncertainty among implementers and companies. In addition, many firms remain unfamiliar with or sceptical about government digital platforms and therefore continue to prefer manual procedures. Together, these factors illustrate how misaligned standards, insufficient resources, weak communication, and hesitant stakeholder attitudes combine to produce process, institutional, and behavioural inefficiencies in SPBE implementation.

These perspectives suggest that SPBE related inefficiencies are not merely technical implementation issues but reflect deeper tensions between policy design, organisational capacity, and contextual realities. At the centre, strong formal authority and clear normative goals coexist with fragmented systems, rigid procedures, and limited behavioural change. At the provincial level, locally relevant innovations emerge, yet they are constrained by resource limitations, uneven understanding of national regulations, and weak political endorsement. Public policy implementation theory therefore helps to explain why a policy that is normatively well specified and supported by a detailed regulatory framework can still result in structural, processual, communicative, and political inefficiencies. This analysis underscores the need to align SPBE objectives, instruments, and standards more closely with implementation capacities and contexts, and to strengthen communication, integration, and sustained leadership if digital government reform is to move beyond formal compliance toward substantive transformation of public service delivery.

### 5.2 Inefficiencies in SPBE implementation

The use of classical public policy implementation theory provides a more comprehensive explanation of why and how inefficiencies arise in the implementation of SPBE. Policy implementation theories clarify why SPBE does not fully realise its normative goals: policy objectives are not fully internalised, implementation structures are fragmented, resources are limited, and the socio political and bureaucratic context is resistant to change. ^20, 21^ From the perspective of public policy implementation theory, the identified inefficiencies are a logical consequence of gaps in goal clarity, instruments, coordination, and political support. At the central level, SPBE is backed by a clear presidential mandate, yet the design of digital attendance systems that do not allow reasonable corrections shows that instruments are not sufficiently adaptive to everyday administrative realities. At the provincial level, limited budgets for socialisation and unequal understanding of SPBE among officials and firms weaken the communication. ^21^

These relationships between theoretical dimensions and empirical findings are summarised in Table 6, which links core concepts from each theoretical strand to concrete observations at Kemnaker RI and the Banten Provincial Manpower Office, and shows how these are translated into structural, process based, communicative, and political forms of inefficiency.

**Table 6.** Comparison between theoretical dimensions and empirical findings.

Theory	Core concepts	Empirical findings	Forms of inefficiency
Mazmanian and Sabatier (top down approach)	Clarity of policy goals and institutional design	SPBE objectives are normatively clear (digital efficiency), but applications are fragmented across institutions.	Overlapping and siloed applications that slow down bureaucratic processes.
Van Meter and Van Horn (policy performance)	Standards, resources, and implementers’ capacity	Formal standards exist (presidential and ministerial regulations), but human and financial resources for socialisation are limited.	Uneven digital service delivery and centre–province implementation gaps.

The synthesis of these perspectives suggests that SPBE generates new inefficiencies not because the policy has failed completely, but because it operates within a fragmented, path dependent, and politically contingent governance environment. ^36^ At the centre, SPBE can be characterised as dense in rules and systems but weak in integration and flexibility; at the provincial level, SPBE appears more innovative and closer to citizen needs, yet constrained by scarce resources, partial integration with national platforms, and reliance on discretionary political support. This implies that reducing SPBE related inefficiencies cannot focus only on issuing new regulations or technical standards, but must also strengthen coordination and learning mechanisms between levels of government, design instruments that are clear yet flexible, and treat local innovations as sources of experimentation that can be scaled and integrated into the national SPBE architecture.

## 6. Conclusion and recommendation

This study examined the implementation of the Electronic-Based Government System (SPBE) in the employment sector by comparing the Ministry of Manpower (Kemnaker) as the central government unit and the Manpower Office of Banten Province as the regional counterpart. Using public policy implementation theory, the findings show that SPBE has formally advanced the digitalisation of public services in line with Presidential Regulation No. 95/2018, as reflected in the use of applications such as *Srikandi* and *e-Presensi* at the national level and SiLoker at the provincial level. However, these achievements remain suboptimal: inefficiencies persist in the form of fragmented and overlapping applications, limited interoperability between central and local systems, rigid digital procedures (e.g. inflexible e-attendance mechanisms), uneven socialisation to users, and the persistence of manual, paper-based bureaucratic practices. This indicates a clear gap between the normative goals of SPBE—efficiency, integration, transparency—and the empirical realities of its implementation.

From a practical perspective, the study recommends strengthening integration between central and local SPBE applications to avoid duplication and ensure interoperability of data and services; designing digital mechanisms that allow fair and transparent corrections so that systems remain credible and adaptable to real-world conditions; allocating sufficient resources for continuous socialisation to citizens and firms supported by structured digital literacy training for civil servants; and stabilising institutional arrangements so that SPBE implementation is anchored in clear organisational procedures and shared performance standards rather than being overly dependent on individual leaders. These steps are essential to reduce structural, process, and communication inefficiencies and to ensure that SPBE genuinely improves the effectiveness and responsiveness of public services in the employment sector.

From a theoretical perspective, the research demonstrates that SPBE implementation is best understood through a synthesis of public policy implementation theory: clear formal objectives and instruments alone are not sufficient when implementation unfolds in a fragmented, multi-level, and politically contingent environment. The findings show how content and context of policy, power relations, resource asymmetries, path dependence, feedback loops, and local innovation jointly shape non-linear outcomes in digital bureaucracy reform. Future studies are encouraged to develop and test adaptive, network-based implementation models for SPBE that explicitly incorporate multi-actor interactions, central–local interdependencies, and mechanisms to manage fragmentation, so that digital government reforms can move closer to achieving integrated, citizen-centred, and learning-oriented public governance.

## Ethical approval

This study involved human participants through in-depth interviews with policymakers, implementers, and service users. Ethical approval was obtained from the Ethics Committee of the Faculty of Administrative Sciences, Universitas Brawijaya (Approval No. 10406/UN10.F0301/B/PP/2025), which also approved the use of verbal informed consent procedures for this study.

## Informed consent

Prior to data collection, all participants received an information sheet explaining the study objectives, interview procedures, expected duration, potential risks and benefits, confidentiality measures, and their right to decline or withdraw at any time without consequences. Verbal informed consent was obtained from all participants before each interview commenced. Verbal consent was selected instead of written consent to minimise administrative burden and reduce potential participant discomfort associated with formal documentation in sensitive governance-related discussions involving policymakers and public service actors. This procedure was reviewed and approved by the Ethics Committee of the Faculty of Administrative Sciences, Universitas Brawijaya under Approval No. 10406/UN10.F0301/B/PP/2025. To protect participant privacy, identifying information was removed from transcripts and all data were stored securely with restricted access.

## Data Availability

SPBE Interview Questions and Anonymized Interview Data. https://doi.org/10.6084/m9.figshare.31360138. ^43^ This project contains the following underlying data:
• SPBE Interview Questions.pdf (Interview question guide for policymakers, implementers, and users). • Data.pdf (Anonymized interview data: English translation/summary excerpts with all identifying details removed). Data are available under the terms of the Creative Commons Attribution 4.0 International license.

## References

[1] Danar OR Novita AA Anggriawan T : Between productivity booster and structural resistance: A trajectory and limit of agile governance. *J. Gov. Regul.* Mar. 2024;13(1, special Issue):341–349. 10.22495/jgrv13i1siart8

[2] Yusifov F : A citizen-centric approach for digital government transformation: The case of Azerbaijan. *Inf. Dev.* Sep. 2025. 10.1177/02666669251370361

[3] Taufiqurokhman T Satispi E Andriyani L : Digital Governance Models in Developing Countries and the Effectiveness of Public Services. *Citiz. Gov. Rev.* 2025;2(2):172–189.

[4] Asmawa Hakim A Hermawan : Transforming Public Policy in Developing Countries: A Comprehensive Review of Digital Implementation. *J. ICT Stand.* Nov. 2024;337–364. 10.13052/jicts2245-800X.1235

[5] Wahyu Sulistya AQ : A Case Study of Indonesian Government Digital Transformation: Improving Public Service Quality through E-government Implementation. *2019 5th International Conference on Science and Technology (ICST).* IEEE;Jul. 2019; pp.1–6. 10.1109/ICST47872.2019.9166234

[6] Aulia TR : Quality Optimisation of Electronic-Based Government Systems (SPBE) Through the Application of the COBIT Framework in Tangerang Regency. *J. Ilm. Adm. Publik.* 2025;11(1):67–80.

[7] Maulana RY Marjamat U Subekti D : Smart Governance Challenges in Indonesian Local Government. *Resilience Through Digital Innovation: Enabling the Twin Transition.* University of Maribor Press;May 2024; pp.33–56. 10.18690/um.fov.4.2024.3

[8] Ovais Ahmad M Markkula J Oivo M : Factors affecting e-government adoption in Pakistan: a citizen’s perspective. *Transform. Gov. People, Process Policy.* May 2013;7(2):225–239. 10.1108/17506161311325378

[9] Shareef MA Kumar U Kumar V : Identifying critical factors for adoption of e-government. *Electron. Gov. an Int. J.* 2009;6(1):70. 10.1504/EG.2009.022594

[10] Baeuo MOM Rahim NZBA Alaraibi AAM : Technology Aspects of E-Government Readiness in Developing Countries: A Review of the Literature. *Comput. Inf. Sci.* Sep. 2016;9(4):1. 10.5539/cis.v9n4p1

[11] Waheduzzaman W Miah SJ : Readiness assessment of e-government: a developing country perspective. *Transform. Gov. People, Process Policy.* Oct. 2015;9(4):498–516. 10.1108/TG-05-2014-0018

[12] Adjei-Bamfo P : An e-government framework for assessing readiness for public sector e-procurement in a lower-middle income country. *Inf. Technol. Dev.* Oct. 2020;26(4):742–761. 10.1080/02681102.2020.1769542

[13] Alomari MK Sandhu K Woods P : Exploring citizen perceptions of barriers to e-government adoption in a developing country. *Transform. Gov. People, Process Policy.* Mar. 2014;8(1):131–150. 10.1108/TG-05-2013-0013

[14] Safiah Maznorbalia A Aiman Awalluddin M : Users Acceptance of E-Government System in Sintok, Malaysia: Applying the UTAUT Model. *Policy Gov. Rev.* Jan. 2021;5(1):66. 10.30589/pgr.v5i1.348

[15] Aldhi IF : Bridging Digital Gaps in Smart City Governance: The Mediating Role of Managerial Digital Readiness and the Moderating Role of Digital Leadership. *Smart Cities.* Jul. 2025;8(4):117. 10.3390/smartcities8040117

[16] Danar OR : Digital transformation of Indonesian administration and bureaucratic system. *Int. J. Electron. Gov.* 2024;16(2):152–171. 10.1504/IJEG.2024.140789

[17] Resmita T Sartika I : Evaluating the SPBE-Based Monitoring and Evaluation System for Village Facilitators’ Performance. *J. Ilm. Multidisiplin Indones.* 2025;4(7):754–762.

[18] Makatur MY Gultom RAG Wadjidi AF : Analysis of Electronic-Based Government Systems (SPBE) Using the Six-Ware Cyber Security Framework (SWCSF) in West Sumatra Province. *Formosa J. Multidiscip. Res.* Feb. 2025;4(2):899–912. 10.55927/fjmr.v4i2.67

[19] Hidayati NN Srimoeljanto A : Knowledge Management for Electronic-Based Government System Using Semantic Thesaurus. *J. Ilmu Komput. dan Inf.* Jul. 2023;16(2):103–113. 10.21609/jiki.v16i2.1148

[20] Sabatier PA Mazmanian DA : The prospects for effective implementation of regulatory policy. *Can Regulation Work?: The Implementation of the 1972 California Coastal Initiative.* Boston, MA: Springer US;1983; pp.1–26.

[21] Van Meter DS Van Horn CE : The Policy Implementation Process. *Adm. Soc.* Feb. 1975;6(4):445–488. 10.1177/009539977500600404

[22] Sabani A : *An empirical examination of e-government adoption in Indonesia.* RMIT University;2024.

[23] Maynard-Moody S Herbert AW : Beyond Implementation: Developing an Institutional Theory of Administrative Policy Making. *Public Adm. Rev.* Mar. 1989;49(2):137. 10.2307/977333

[24] Sager F Gofen A : The polity of implementation: Organizational and institutional arrangements in policy implementation. *Governance.* Apr. 2022;35(2):347–364. 10.1111/gove.12677

[25] Homburg V : *Understanding e-government: Information systems in public administration.* Routledge;2008.

[26] Hung S-Y Chen K Su Y-K : The effect of communication and social motives on E-government services through social media groups. *Behav. Inform. Technol.* Jul. 2020;39(7):741–757. 10.1080/0144929X.2019.1610907

[27] Okan AA : Exploring the Landscape of e-Government Maturity Models: Insights from Systematic Mapping Study and Comparative Analysis. *Digit. Gov. Res. Pract.* Jun. 2024;5(2):1–26. 10.1145/3656586

[28] Zhang Y Kimathi FA : Exploring the stages of E-government development from public value perspective. *Technol. Soc.* May 2022;69:101942. 10.1016/j.techsoc.2022.101942

[29] Hazineh SA Eleyan D Alkhateeb M : E-government: Limitations and challenges: A general framework for to consider in both developed and developing countries. *Int. J. Sci. Technol. Res.* 2022;11(1).

[30] Mahlangu G Ruhode E : Factors enhancing e-government service gaps in a developing country context. 2021.

[31] Apriliyanti ID Kusumasari B Pramusinto A : Digital divide in ASEAN member states: analyzing the critical factors for successful e-government programs. *Online Inf. Rev.* Mar. 2021;45(2):440–460. 10.1108/OIR-05-2020-0158

[32] Permana D : Dynamics of Public Policy in the Digital Era: A Case Study of e-Government Implementation in Indonesia. *Influ. Int. J. Sci. Rev.* 2023;5(3):163–174.

[33] Sundari W Sartika I : Advancing Public Service Quality through Indonesia’s Electronic-Based Government System. *J. Ilm. Multidisiplin Indones.* 2025;4(8):902–911.

[34] Republik Indonesia: Peraturan Presiden Republik Indonesia Nomor 95 Tahun 2018 tentang Sistem Pemerintahan Berbasis Elektronik [Presidential Regulation of the Republic of Indonesia Number 95 of 2018 on the Electronic-Based Government System]. 2018.

[35] Kementerian Pendayagunaan Aparatur Negara dan Reformasi Birokrasi: Peraturan Menteri Pendayagunaan Aparatur Negara dan Reformasi Birokrasi Nomor 5 Tahun 2020 tentang Pedoman Manajemen Risiko Sistem Pemerintahan Berbasis Elektronik [Regulation of the Minister of Administrative and Bureaucratic Reform Number 5 of 2020 on G]. 2020.

[36] Rahma Aulia V : Mapping SPBE Challenges in Indonesia: Systematic Review by Architecture Domain. *J. Komput. Teknol. Inf. Sist. Inf.* Jun. 2025;4(1):174–183. 10.62712/juktisi.v4i1.368

[37] Priya A : Case Study Methodology of Qualitative Research: Key Attributes and Navigating the Conundrums in Its Application. *Soc. Secur. Bull.* Jan. 2021;70(1):94–110. 10.1177/0038022920970318

[38] Miles MB Huberman AM Saldaña J : Qualitative data analysis: A methods sourcebook. 2014.

[39] Muzaqqi F Fitrianto H : Comparison of e-government acceleration in five regions: Case studies following the issuance of Presidential Regulation 95/2018. *Masyarakat, Kebud. dan Polit.* May 2023;36(2):230–245. 10.20473/mkp.V36I22023.230-245

[40] Agusmidah Shalihah F : Indonesian migrant workers: a socio-economic analysis with regard to the integrated services practice implementation. *Econ. Ann.* Jun. 2023;203(5–6):70–75. 10.21003/ea.V203-08

[41] Asianto A Fatonah NS Firmansyah G : Journal Series on Governance and Management of IT in Electronic-Based Government Systems (SPBE) in Indonesia. *J. Indones. Sos. Sains.* Sep. 2023;4(09):802–814. 10.59141/jiss.v4i09.880

[42] Arfan S Mayarni M Nasution MS : Responsivity of Public Services in Indonesia during the Covid-19 Pandemic. *Budapest Int. Res. Critics Inst. Humanit. Soc. Sci.* Jan. 2021;4(1):552–562. 10.33258/birci.v4i1.1638

[43] Yulianto T Saleh C Noor I : SPBE Interview Questions and Anonymized Interview Data. 2026. 10.6084/m9.figshare.31360138

