# Supplementary material for: Policy Implementation in the Digital Era: A Comprehensive Analysis of the Electronic-Based Government System (SPBE) in Indonesia
Source: F1000Res. 2026 Apr 15;15:523. [Version 1] doi: 10.12688/f1000research.175463.1 (PMC13153765; doi:10.12688/f1000research.175463.1)
Supplement: Peer review history

# Reviewer report: Reviewer response for version 1

**DOI:** 10.5256/f1000research.193446.r481273

**Published:** 2026-05-07

**Authors:** Eka Desy Purnama

**Affiliations:** 1 Krida Wacana Christian University, Jakarta, Jakarta, Indonesia

This article addresses an important and timely issue in digital governance and public policy implementation, particularly the implementation of Indonesia’s Electronic-Based Government System (SPBE) in the labour sector. Overall, the manuscript is relevant, well-structured, and supported by an appropriate qualitative case study design. The comparison between central and provincial implementation provides useful empirical insights into fragmentation, interoperability gaps, and coordination challenges in SPBE implementation. However, although the article is generally acceptable, several revisions are still needed to strengthen its methodological clarity, conceptual precision, and overall contribution.

**Suggested Revisions:**

1. **Methodological transparency**
  The methodological explanation should be made clearer, particularly regarding how the data were collected, analysed, and used to support the findings.
2. **Theoretical contribution**
  The manuscript would benefit from deeper engagement with recent digital governance literature, beyond classical public policy implementation theories, to better position its contribution within current scholarly debates.
3. **Clarify key concepts and terminology**
  The use of several terms still appears inconsistent in some parts of the article, particularly the term SPBE. The authors should review the entire article and ensure that key terms are used consistently throughout the article.
4. **Conclusion, limitations, and future research direction**
  The limitations and future research agenda should be stated more explicitly and linked more directly to the study’s empirical scope, especially its focus on one sector and two institutional settings.

The manuscript is suitable for further consideration, but it would benefit from targeted revisions to improve its scholarly rigor and presentation. The required revisions are manageable and do not undermine the overall value of the study. Therefore, I recommend Approved with Reservations, with the expectation that the authors address the suggested improvements before the article proceeds further.

Is the work clearly and accurately presented and does it cite the current literature?

Partly

If applicable, is the statistical analysis and its interpretation appropriate?

Not applicable

Are all the source data underlying the results available to ensure full reproducibility?

Yes

Is the study design appropriate and is the work technically sound?

Yes

Are the conclusions drawn adequately supported by the results?

Yes

Are sufficient details of methods and analysis provided to allow replication by others?

Yes

Reviewer Expertise:

Human Resource Management, Financial Management, Operations Management, Marketing Management, Knowledge Management, and Applied Economics.

I confirm that I have read this submission and believe that I have an appropriate level of expertise to confirm that it is of an acceptable scientific standard, however I have significant reservations, as outlined above.

# Reviewer report: Reviewer response for version 1

**DOI:** 10.5256/f1000research.193446.r481268

**Published:** 2026-05-06

**Authors:** Muhammad Khairul Anwar

**Affiliations:** 1 Universitas Negeri Surabaya, Surabaya, Indonesia

The article addresses an important and timely topic concerning the implementation of the Electronic-Based Government System (SPBE) in Indonesia, particularly in the labour sector.

 Overall, the article is clearly structured, relevant to the field of public administration and digital governance, and supported by an appropriate qualitative case study design. The use of public policy implementation theory helps explain why SPBE implementation may still produce fragmentation, limited interoperability, rigid procedures, and uneven adoption despite having a clear regulatory foundation. The conclusions are generally consistent with the findings and provide relevant practical recommendations for improving integration, coordination, socialisation, and digital service governance.

 I consider the article suitable for approval. However, several minor revisions would further improve the clarity, consistency, and academic contribution of the study.

**Minor comments for revision**

1. **Improve consistency in terminology**
  The article uses several related but distinct terms, including “e-government,” “digital government,” “digital transformation,” “digital bureaucracy,” and “SPBE.” The authors should briefly clarify the conceptual relationship among these terms, particularly how SPBE is positioned as Indonesia’s formal policy framework within the broader digital government and digital transformation agenda.
2. **Clarify the methodology in the Abstract.** The methodology in the abstract is still described rather generally and needs to be explained more clearly so that readers can better understand how the study was conducted.
3. **Future research direction. **The future research recommendation is relevant but still too broad. The authors should make it more specific and directly linked to the study’s empirical findings, such as central–local SPBE integration, interoperability, user adoption, or comparative studies across provinces/sectors.
4. **Add limitations section or statement.** The article would be strengthened by explicitly acknowledging its limitations. Since the study uses a qualitative case study design and focuses on the labour sector, the generalisability of the findings may be limited. A clearer limitations statement would improve the transparency and methodological balance of the article.

Is the work clearly and accurately presented and does it cite the current literature?

Yes

If applicable, is the statistical analysis and its interpretation appropriate?

Not applicable

Are all the source data underlying the results available to ensure full reproducibility?

Yes

Is the study design appropriate and is the work technically sound?

Yes

Are the conclusions drawn adequately supported by the results?

Yes

Are sufficient details of methods and analysis provided to allow replication by others?

Yes

Reviewer Expertise:

Public Administration, Policy, and Governance

I confirm that I have read this submission and believe that I have an appropriate level of expertise to confirm that it is of an acceptable scientific standard.

# Reviewer report: Reviewer response for version 1

**DOI:** 10.5256/f1000research.193446.r475839

**Published:** 2026-04-21

**Authors:** Rakibul Hasan Chowdhury

**Affiliations:** 1 Trine University, Angola, Indiana, USA

**Summary of the Article**

 This article examines the implementation of Indonesia’s Electronic-Based Government System (SPBE) using a qualitative, multi-level case study approach. The study focuses on two administrative levels: the Ministry of Manpower (central level) and the Banten Provincial Manpower Office (subnational level). Drawing on classical frameworks from Public Policy Implementation, the authors analyze how SPBE is interpreted and enacted by policy-makers, implementers, and users.

 The findings indicate that while SPBE has accelerated digitalization and shifted administrative practices from manual to digital systems, implementation remains fragmented. Key challenges include limited interoperability, uneven institutional capacity, rigid procedures, and insufficient coordination between central and local governments. The study concludes with policy recommendations emphasizing integration, user-centered design, and improved governance mechanisms.

**Evaluation and Justification of Responses**

**1. Clarity of Presentation and Use of Literature – *Partly***

 The manuscript is generally well-structured and clearly written, with a logical flow from introduction to conclusion. The use of literature on e-government and policy implementation is adequate and relevant.

 However, the engagement with **recent and advanced literature** remains somewhat limited. The manuscript relies heavily on classical implementation theories and does not sufficiently incorporate newer developments in digital governance, such as:

- Platform governance
- Adaptive or network governance models
- AI-enabled public administration

**Required improvements:**

- Integrate more recent (2022–2025) high-impact studies in digital governance
- Strengthen the theoretical positioning beyond classical frameworks
- Provide clearer differentiation of this study’s contribution relative to existing SPBE literature

**2. Study Design and Technical Soundness – *Yes***

 The qualitative case study design is appropriate for exploring complex, context-dependent policy implementation processes. The selection of:

- Central and provincial institutions
- Multiple actor groups (policy-makers, implementers, users)

is methodologically sound and enhances analytical depth.

 The use of:

- Interviews
- Observations
- Document analysis

demonstrates strong triangulation and supports the credibility of findings.

**3. Methodological Detail and Replicability – *Partly***

 While the general methodology is clearly described, **important details are missing**, which limits reproducibility.

**Key gaps include:**

- Number of participants interviewed
- Sampling size justification
- Duration of data collection
- Detailed explanation of coding and analysis procedures
- Whether qualitative analysis software (e.g., NVivo) was used

**Required improvements (critical for scientific soundness):**

- Clearly state the number and categories of participants
- Describe the coding framework and analytical process step-by-step
- Explain how thematic saturation was achieved
- Provide more transparency on data interpretation and validation

**4. Statistical Analysis – *Not Applicable***

 The study is qualitative and does not involve statistical analysis. Therefore, this criterion is not applicable.

**5. Availability of Source Data – *Partly***

 The authors provide access to anonymized interview data and research instruments via an open repository, which is commendable and aligns with open science principles.

 However, **full reproducibility is limited** because:

- Raw transcripts are not fully available (only summarized/translated excerpts)
- Contextual metadata is limited
- Observational data is not fully documented

**Suggested improvements:**

- Provide more detailed anonymized transcripts (where ethically feasible)
- Clarify the structure and completeness of shared datasets
- Include coding frameworks or analytical matrices as supplementary material

**6. Validity of Conclusions – *Yes***

 The conclusions are consistent with the empirical findings and are logically derived from the analysis. The identification of:

- Fragmentation
- Interoperability gaps
- Institutional and behavioral constraints

is well supported by the data.

 The policy recommendations are relevant and grounded in the findings. However, their impact could be strengthened by deeper analytical explanation of causal mechanisms.

**Key Issues That Must Be Addressed**

 To ensure the manuscript is scientifically robust and suitable for indexing, the following issues should be addressed:

1. **Enhance methodological transparency**
  - Provide detailed information on sample size, data collection, and analysis procedures
2. **Strengthen theoretical contribution**
  - Engage with contemporary digital governance literature beyond classical models
3. **Clarify and operationalize key concepts**
  - Particularly the concept of “inefficiency”
4. **Improve data transparency**
  - Expand the availability and clarity of underlying qualitative data

**Additional Suggestions (Recommended but Not Mandatory)**

- Reduce descriptive repetition in the Results section
- Strengthen comparative discussion with other developing countries
- Improve clarity and conciseness of tables
- Conduct minor language editing for readability

**Final Assessment**

 This study addresses an important and timely topic in digital governance and provides valuable empirical insights into SPBE implementation. With revisions focused on methodological rigor, theoretical depth, and data transparency, the manuscript has strong potential to make a meaningful contribution to the field.

Is the work clearly and accurately presented and does it cite the current literature?

Partly

If applicable, is the statistical analysis and its interpretation appropriate?

Not applicable

Are all the source data underlying the results available to ensure full reproducibility?

Partly

Is the study design appropriate and is the work technically sound?

Yes

Are the conclusions drawn adequately supported by the results?

Yes

Are sufficient details of methods and analysis provided to allow replication by others?

Partly

Reviewer Expertise:

Digital Governance, E-Government, Public Policy Implementation, Digital Transformation, Artificial Intelligence in Public Administration, Data Analytics, and Enterprise Innovation Systems

I confirm that I have read this submission and believe that I have an appropriate level of expertise to confirm that it is of an acceptable scientific standard, however I have significant reservations, as outlined above.
